# Clinical evaluation of a dedicated next generation sequencing panel for routine glioma diagnostics

**DOI:** 10.1186/s40478-018-0633-y

**Published:** 2018-11-23

**Authors:** Nathalie E. Synhaeve, Martin J. van den Bent, Pim J. French, Winand N. M. Dinjens, Peggy N. Atmodimedjo, Johan M. Kros, R. Verdijk, Clemens M. F. Dirven, Hendrikus J. Dubbink

**Affiliations:** 1 Department of Neurology, Elisabeth Tweesteden Hospital, Tilburg, The Netherlands; 2 Department of Neurology, Brain Tumor Center at Erasmus MC Cancer Institute, University Medical Center Rotterdam, PO Box 5201, 3008AE Rotterdam, The Netherlands; 3 Department of Pathology, Brain Tumor Center at Erasmus MC Cancer Institute, Rotterdam, The Netherlands; 4 Department of Neurosurgery, Brain Tumor Center at Erasmus MC Cancer Institute, Rotterdam, The Netherlands

**Keywords:** Glioma, Molecular diagnostics, Next-generation-sequencing

## Abstract

Since 2013 next-generation sequencing (NGS) targeting genes mutated in diffuse gliomas is part of routine diagnostics in our institute. In the present report, we evaluate the use of this custom tailored NGS platform on 434 samples. The NGS panel assesses mutations in *ATRX, CIC, EGFR, FUBP1, NOTCH1, PTEN; H3F3A, IDH1/2, PIK3CA,* and *BRAF*, amplifications in *EGFR* or *MDM2* and copy number alterations (CNA) of chromosome 1p, 7, 10 and 19q. *TERT* promoter mutations were assessed separately when indicated. Of the 433 samples of individual tumors with NGS data available, 176 cases were diagnosed as grade 2 or 3 glioma (40.6) and in 201 patients a glioblastoma (46.4%). Of the remaining 56 patients, 22 had inconclusive histology. In 378 cases (87.1%) a diagnosis solely based on glioma-targeted NGS could be established and resulted in a different diagnosis in ~ 1/4 of the cases. In 17 out of 22 cases without a conclusive histological diagnosis NGS resulted in a molecular diagnosis.

The current study on a large cohort of patients confirms the diagnostic strength of the platform we developed, with a clear separation of glioma subgroups with different outcomes. It demonstrates the diagnostic value and the efficiency of glioma-targeted NGS for routine glioma diagnostics allowing with a single assay a glioma diagnosis in the large majority of cases. It allows in one run the molecular assessments required for the WHO classification of diffuse gliomas, including the recent recommendations to assess copy number alterations of chromosome 7 and 10, and of the *TERT* promoter region in *IDH*wt lower grade glioma.

**Electronic supplementary material:**

The online version of this article (10.1186/s40478-018-0633-y) contains supplementary material, which is available to authorized users.

## Introduction

In 2016 a major revision of the WHO classification for tumors of the central nervous system was implemented [6]. The main adjustment was the incorporation of molecular criteria to the diagnostic classification. In adult diffuse glioma this is now centered around isocitrate dehydrogenase (*IDH*) and 1p/19q diagnostics. However, several other markers are potentially relevant for routine testing, including assessment of copy number alterations (CNA) of chromosome 7 and 10, (homozygous) *CDKN2A* loss, and mutations in the *TERT* promoter or *BRAF* and *H3F3A* genes [14, 20]. Previous studies have shown that molecular characteristics do not only hold a diagnostic value, but also can give more detailed information about prognosis. An example is the significant worse overall survival of patients with a grade II glioma with an *IDH-*wildtype tumor with gain of chromosome 7 and loss of chromosome 10 or with TERT mutations compared to those with an *IDH*-wildtype tumor without these lesions [1, 13, 16, 18, 21]. Another study also identified PI3-kinase mutations as markers of poor prognosis in *IDH*-mutated and *ATRX*/*TP53* mutated diffuse gliomas, median survival 3.7 v. 6.3 years (*P* = 0.02) [7].

Since 2013, in our institute a next-generation sequencing (NGS) panel targeting genes frequently mutated in gliomas is part of routine diagnostics. We previously showed that this approach identified clinically relevant glioma subgroups by analysis of historical samples obtained in the EORTC trial on PCV chemotherapy of anaplastic oligodendroglial tumors, with a very low failure rate [8]. We also demonstrated that the use of single nucleotide polymorphisms (SNP’s) in this panel allows the reliable assessment of CNA’s [9]. In the present report we evaluate retrospectively the routine use of this custom tailored NGS platform in everyday clinical practice, to assess whether it allows us to diagnose patients accurately and efficiently.

## Materials and methods

We included all patients from whom glioma-targeted NGS data were available between 2013 and March 17th 2017, during which period all findings were routinely entered into a database. As part of the present evaluation of this diagnostic platform, we added clinical, demographic and survival information. Since many patients were referred from other institutions with limited data on the clinical course, the date of the first (diagnostic) surgery was used as the date of diagnosis and survival was measured from this date. The principle aims of this study were to describe and evaluate the routine use and additional value of glioma targeted NGS, with emphasis on cases without a histological diagnosis or without a molecular diagnosis, on histological grade II and III *IDH* wild type glioma.

In the first phase after introduction of the targeted NGS panel, the platform was routinely used for all grade II and III gliomas, all cases with oligodendroglial histology, patients with a diagnosis of a glioblastoma below the age of 51, all diagnostic challenging cases and cases in which the histological diagnosis was reviewed. Following the introduction of the WHO 2016 criteria, the upper age limit for routine testing of glioblastoma was increased to 55 years of age and currently includes immunohistochemistry for IDH R132H mutations in glioblastoma patients over that age [15, 23]. In addition, because of actively recruiting clinical trials in glioblastoma with either amplification of *EGFR, MET*, or *MDM2* or mutations in *PTEN,* or *BRAF,* patients potentially eligible for these trials were also investigated. *TERT* promoter mutations were assessed separately in cases in which it was considered clinically indicated (grade 2 and 3 astrocytoma *IDH*wt) using SNAPSHOT analysis [11] as described previously. The used NGS panel assesses mutations in *ATRX, CIC, EGFR, FUBP1, NOTCH1, PTEN; H3F3A, IDH1/2, PIK3CA* and *BRAF*; amplifications in *EGFR, MDM2* and *MET* and CNA’s of chromosome 1p, 19q, 10 and 7 as described elsewhere [8, 9, 16]. Analysis of *MDM2* and *MET* amplification were added later once trials were activated that required these as inclusion criteria, and after validation of the assay by FISH. The limit of detection of SNP analysis for loss of heterozygosity determination has been shown to be approximately 20% of tumor cells [9]. The panel was further modified in March 2017 to include more SNP’s for assessment of additional CNA’s (incl 9p, 17) and mutations more relevant for childhood brain tumors. No patients tested after this modification of the panel were included in the present series.

Criteria for the molecular diagnosis of diffuse glioma were defined as follows:

- molecular astrocytoma: *IDH* mutation, without 1p/19q co-deletion.
- molecular oligodendroglioma: *IDH* mutation with 1p/19q co-deletion
- molecular glioblastoma: *IDH1/2* wildtype with either *TERT* promoter mutation, 7+/10- or *EGFR* amplification
- Midline and hemispheral tumors with *H3F3A* mutations: *H3F3A* mutation, subdivided in *H3F3A* K27 M and *H3F3A* G34 M mutated tumors based on the specific mutation present.
- *BRAF*-mutated tumors (although not a clinical entity they are reported separately in view of potential treatment implications)

The panel is not designed to identify fusion genes (eg, the BRAF-KIAA fusion gene). The molecular diagnosis is only made in the presence of positive findings with the NGS panel, not on histology. Survival plots were made for cumulative mortality of all diagnostic groups. Histologically diagnosed glioblastoma were stratified in 2 molecular subgroups based on the presence or absence of *IDH1/2* mutation. Patients who did not reach an endpoint before follow-up ended were censored based on the date they were last seen. Logrank was used to compare survival between groups; a *p*-value below 0.05 was considered significant. The statistical analysis was done using SPSS 24 for Windows.

## Results

In total we included 441 NGS samples (March 2013 – March 2017), from 432 patients. In 9 patients two samples were obtained at different time points. One patient with an oligodendroglioma developed a second tumor outside the field of the primary lesion, which appeared on imaging more consistent with a glioblastoma. NGS confirmed the presence of two different entities (1 oligodendroglioma and 1 glioblastoma). Both lesions from this patient were included in the analysis. In none of the other 8 patients NGS of a sample obtained at progression resulted in a reclassification of the tumor compared to analysis of the earlier sample. The following results are based on the 433 NGS samples of individual tumors.

In 176 out of 433 cases (40.6%) there was a histological diagnosis of a grade 2 or 3 tumor and in 201 patients a glioblastoma (46.4%), Table 1 details the histological findings. In 377 out of 433 cases (87.1%) a diagnosis solely based on the molecular diagnostics could be established (Table 2). In 17 of 22 cases (5.1%) without a conclusive histological diagnosis NGS resulted in a molecular tumor diagnosis (77.3%): 3 were diagnosed with oligodendroglioma (13.6%), 2 with astrocytoma (9.1%), 11 with glioblastoma (50.0%) and 1 with a *BRAF*-mutated tumor (4.5%). Moreover, in 8 of these 22 cases the pathologist did not find evidence of a tumor, whereas the NGS panel found a glioblastoma in 4 patients, an oligodendroglioma in 1 patient and a *BRAF*-mutated tumor in 1 patient (Additional file 1: Figure S1 and Additional file 2: Figure S2). In the 2 remaining cases in which the pathologist did not find evidence of a tumor no molecular aberrations were detected and no conclusive evidence for a tumor was obtained. One of these cases was diagnosed with an autoimmune encephalitis and treated accordingly, the other is lost to follow-up. In 15 out of 433 cases (3.5%) a *BRAF*-mutation was found and in 12 out of 433cases (2.8%) an *H3F3A*-mutated tumor (K27 M: 9 (2.1%), G34 M: 3 (0.7%)). In 123 out of 433 cases (28.4%) molecular characterization led to a change of diagnosis (without taking tumors with *BRAF* or *H3F3A* mutations into account). Additional file 3: Table S1 presents an overview of the histological diagnoses and the molecular diagnosis that were made in each histopathological category.

**Table 1:** Histological diagnosis

	Number of cases (%)
Inconclusive	22 (5.1)
Astrocytoma	83 (19.2)
Anaplastic astrocytoma	38 (8.8)
Oligoastrocytoma	5 (1.2)
Anaplastic oligoastrocytoma	6 (1.4)
Oligodendroglioma	30 (6.9)
Anaplastic oligodendroglioma	14 (3.2)
Glioblastoma	201 (46.4)
Ganglioglioma	6 (1.4)
Pilocytic astrocytoma	9 (2.1)
Other	19 (4.1)
Total	433

**Table 2:** Molecular diagnosis

	Number of cases (%)
No mutations identified	19 (4.4)
Unclassifying mutations	37 (8.5)
Astrocytoma	117 (27.0)
Oligodendroglioma	54 (12.5)
Glioblastoma	179 (41.3)
*H3F3A* K27 M-mutated tumor	9 (2.1)
*H3F3A* G34 M-mutated tumor	3 (0.7)
*BRAF*-mutated tumor	15 (3.5)
Total	434

Out of 19 cases (4.4%) in which no mutations or CNA at all were detected by NGS analysis, there was a histopathological diagnosis in 15 cases (78.9%) of which 4 were pilocytic astrocytoma (including 1 pilomyxoid astrocytoma), 4 astrocytoma, 2 ependymoma, 1 oligodendroglioma, 1 neurocytoma, 1 medulloblastoma, 1 ganglioglioma and 1 desmoplastic infantile astrocytoma; in 1 case histological evaluation revealed only radionecrosis.

In Table 3 an overview can be seen of the prevalence of *TP53, FUBP1, CIC, ATRX, PTEN, NOTCH1* and *PIK3CA* mutations specified for each molecular diagnosis. As expected, *ATRX* and *TP53* mutations were seen in most astrocytomas, and *CIC* and *FUBP1*-mutation were observed in oligodendrogliomas.

**Table 3:** Overview of prevalence of specific mutations specified for each molecular diagnosis

Molecular diagnosis	*TP53*	*FUBP1*	*CIC*	*ATRX*	*PTEN*	*NOTCH1*	*PIK3CA*
	n (%)	n (%)	n (%)	n (%)	n (%)	n (%)	n (%)
Astrocytoma	114 (97.4)	1 (0.9)	3 (2.6)	85 (72.6)	6 (5.1)	9 (7.7)	1 (0.9)
Oligodendroglioma	3 (5.6)	15 (27.8)	26 (48.1)	2 (3.7)	5 (9.3)	7 (13.0)	3 (5.6)
Glioblastoma	45 (25.1)	0 (0)	7 (3.9)	6 (3.4)	81 (45.3)	8 (4.5)	2 (1.1)
*H3F3A* K27 M-mutated tumor	7 (77.8)	1 (11.1)	0 (0)	1 (11.1)	1 (11.1)	1 (11.1)	1 (10.0)
*H3F3A* G34 M-mutated tumor	3 (100)	0 (0)	1 (33.3)	3 (100)	0 (0)	0 (0)	0 (0)
*BRAF*-mutated tumor	1 (6.7)	1 (6.7)	1 (6.7)	0 (0)	0 (0)	1 (6.7)	1 (6.7)
Unclassifying mutations	10 (27.0)	1 (2.7)	1 (2.7)	7 (18.9)	8 (21.6)	3 (8.1)	2 (5.4)

**Fig. 1:**
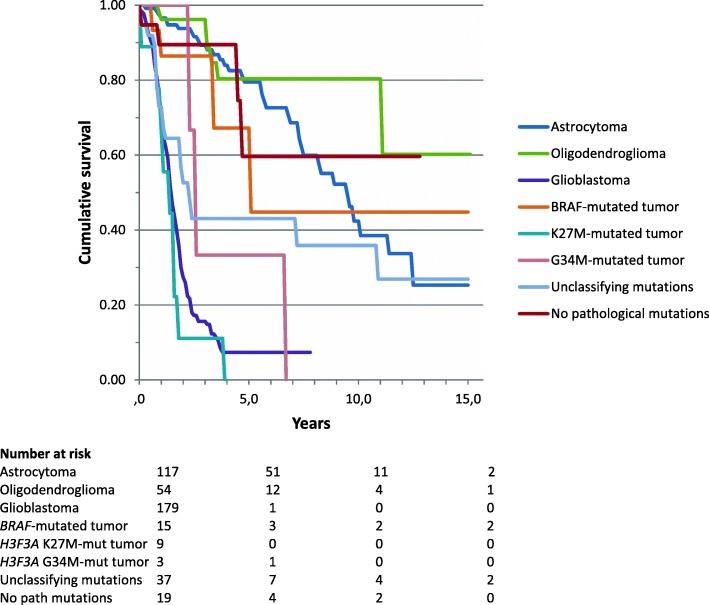
Survival curves for all patients based on the molecular diagnosis

**Fig. 2:**
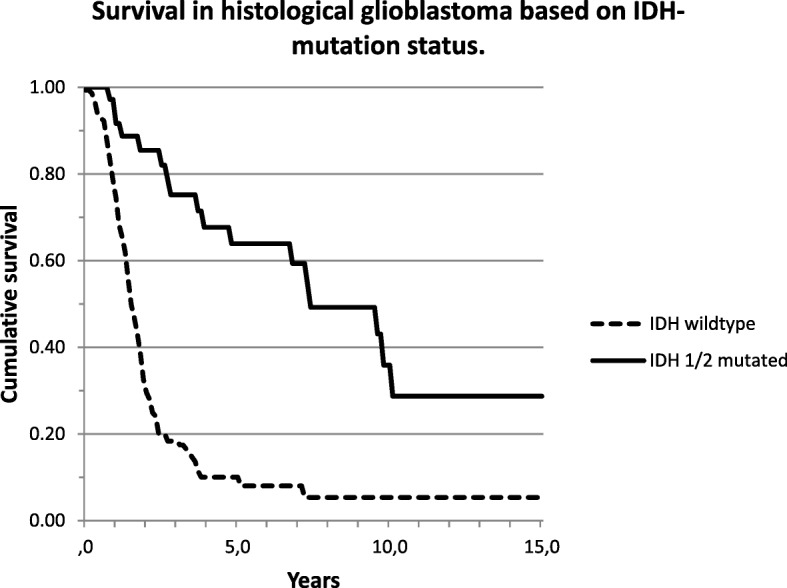
Survival curves for all histological glioblastoma based on *IDH* mutation status

**Fig. 3:**
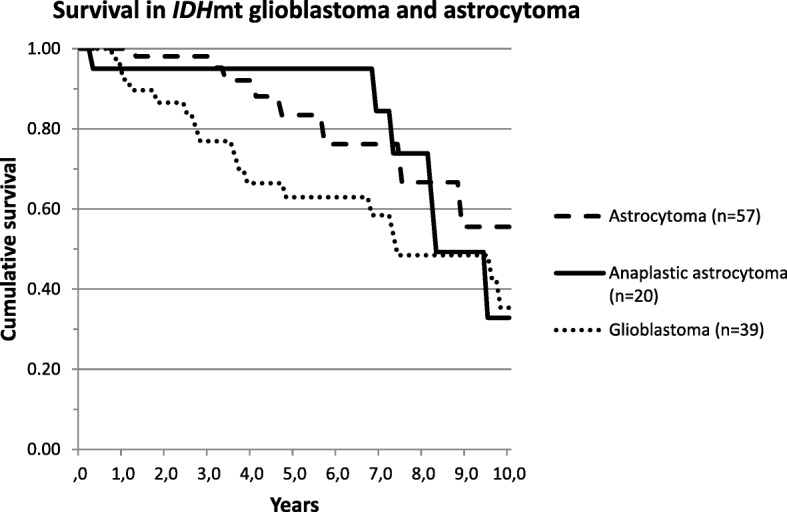
Survival curves for *IDH*mt tumor based on histological diagnosis (astrocytoma, anaplastic astrocytoma or glioblastoma)

Of the 433 patients analyzed, 231 (53.3%) had died at the time of this analysis. Survival based on molecular diagnosis showed a median survival of 9.6 years of patients with astrocytoma, 15.1 years of patients with oligodendroglioma, 1.5 years of patients with glioblastoma, 5.2 years of patients with *BRAF*-mutated tumors, 1.5 years of patients with *H3F3A* K27 M-mutated tumors (*n* = 9) and 2.7 years of patients with *H3F3A* G34 M-mutated tumors (*n* = 3) (Fig. 2). Patients without any detectable mutation (*n* = 19/433) had a median overall survival of 12.8 years in contrast to 2.4 years median survival of patients with mutations that did however not allow a classification (*n* = 37) (Fig. 1). In tumors that were histologically diagnosed as glioblastoma, *IDH*wt tumors (molecular glioblastoma) had a significantly worse survival in comparison to *IDH*mt tumors (median overall survival 1.6 vs 7.5 years; logrank *p* < 0.001) (Fig. 2). For this comparison we excluded 3 out of 201 histological glioblastoma which were reclassified as oligodendroglioma and 7 which were reclassified as *H3F3A*-mutated tumors. Patients with a histologically diagnosed astrocytoma *IDH*mt had a median overall survival of 13.7 years; those with an anaplastic astrocytoma *IDH*mt 8.4 years and glioblastoma *IDH*mt 7.5 years (logrank 0.038 for the difference between astrocytoma and glioblastoma) (Fig. 3).

**Fig. 4:**
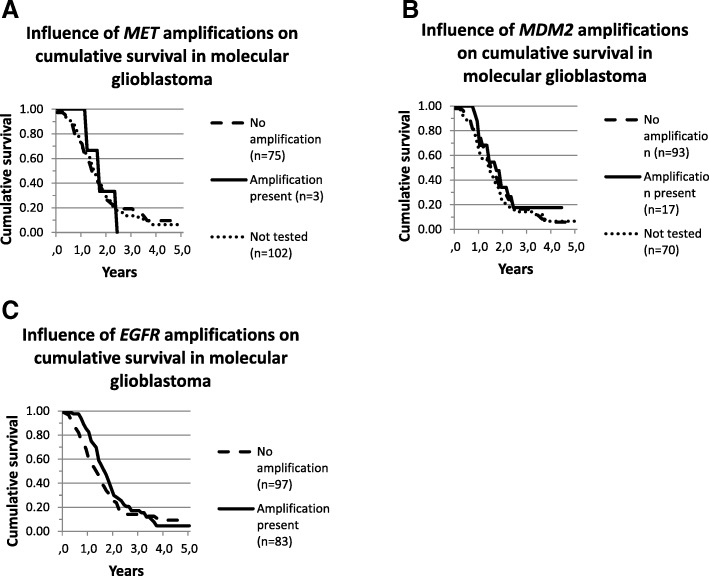
Survival curves for molecular glioblastoma based on (**a**) *MET*, (**b**) *MDM2* and (**c**) *EGFR* amplification status

*MET* and MDM2 amplifications were seen in 3 out of 78 (3.8%) and 17 out of 110 cases (15.5%) molecular glioblastomas respectively tested for these amplifications. *EGFR* amplifications were observed in 83 out of the 179 cases (46.4%). Survival analysis did not show a distinct survival difference between molecular glioblastoma patients with or without *MET*, *MDM2* or *EGFR* amplifications (logrank *p* = 0,997; *p* = 0,478; *p* = 0.181 respectively) (Fig. 4).

## Discussion

This evaluation of our molecular data confirms the great value of glioma targeted NGS for routine brain tumor diagnostics. Our Ion Torrent based NGS panel allows simultaneous assessment of a number of mutations and CNA that are relevant for gliomas. We introduced this platform into routine diagnostics once a validation series showed an improved classification and prognostication of the NGS panel in comparison to classical histopathology, which finding has been confirmed by other series [10, 19, 22]. The improved classification of gliomas using molecular criteria resulted in to the major 2016 revision of the WHO criteria for brain tumors [6]. The current study on a large cohort of patients underscores the diagnostic strength of the platform we developed, with a clear separation of glioma subgroups with different outcomes consistent with our findings in the earlier study on EORTC study 26,951 [8].

Although results of NGS in diffuse glioma have been published before, none of these other studies showed data on the diagnostic value of the NGS panel in cases in which no diagnosis could be established based on the histological findings, nor did they include survival information. Several studies found a high sensitivity of NGS panels to detect genetic alterations known to be present by conventional techniques [17, 19, 22]. Zacher et al. also used a 20-gene panel for an integrated histological and molecular diagnosis of 111 diffuse gliomas, allowing reclassification of oligoastrocytoma and glioblastoma by *IDH*-status and identification of tumors with *H3F3A* mutations [22]. Ballester et al. used a more extensive NGS panel (46–50 genes) in 381 brain tumors and found that the most clinically relevant genes for brain tumor classification in their panel were *IDH1, IDH2, TP53, PIK3CA, BRAF, EGFR, PDGFRA* and *FGFR1/2/3*. Sahm et al. [12] found that information derived from their NGS protocol identified potential targets for experimental therapy (i.e. EGFR, BRAF, PTEN in 37/47 (79%) glioblastomas, 9/10 (90%) pilocytic astrocytomas, and 5/14 (36%) medulloblastomas in a prospective cohort (*n* = 71) [22]. This is in line with our results, although it is fair to say that at present only BRAF V600E mutations represent a validated target for precision medicine (Table 3).

A major advantage of a glioma-targeted NGS approach like this panel is the simultaneous detection of several markers relevant for glioma diagnostics, including copy number alterations, allowing glioma diagnostics according to the revised WHO 2016 classification. These can each be individually assessed by other tests (eg, immunohistochemistry, FISH, Sanger sequencing) and then usually carried out sequentially but that makes the diagnostic process more time consuming. Although NGS may be a relatively expensive diagnostic method, it yields with one assay information that otherwise would require several tests. Moreover, the costs of NGS are rapidly decreasing making it more affordable. Also, the test can be done on very limited amounts of tissue (minimal requirement is 1 ng of DNA from approximately 150 cells consisting of at least 30% neoplastic cells), independent of the method by which the tissue has been obtained; ie. resection, biopsies or even cytology.

The routine assessment of potentially actionable mutations that may have implications for prognosis and treatment is another argument for the routine use of NGS in glioma. *BRAF* mutations are in particular interesting considering their potential treatment implications. Although *BRAF* mutations are no part of the WHO classification and not tumor specific, they are present in certain glioma subtypes with an increased rate and have potentially clinical implications since drugs are available that are active against some *BRAF* mutations (in particular the BRAF V600E mutation). We have been taken by surprise in several cases where despite a histological diagnosis without an increased likelihood of a *BRAF* mutation a BRAF V600E mutation was identified. Another major advantage is the further classification of grade II and III *IDH*wt astrocytomas, of which some have molecular features that allow them to be classified as glioblastoma, holding prognostic and treatment implications [2, 14]. The recently published 3rd paper of the cIMPACT-NOW committee for the integration of new information into the classification of brain tumors now recommends to classify *IDH*wt grade II or III astrocytoma with either high level *EGFR* amplification, or whole chromosome 7 gain in combination with whole chromosome 10 loss, or *TERT* promoter mutation as ‘diffuse astrocytic glioma, *IDH*-wildtype, with molecular features of glioblastoma, WHO grade IV’ [4]. This is similar to the criteria we used for the diagnosis ‘molecular glioblastoma’. Vice versa, our data confirm the clear difference in prognosis between *IDH*mt and *IDH*wt glioblastoma. The median OS in our *IDH*mt glioblastoma possibly reflects a bias towards performing NGS in glioblastoma patients with an unusual long survival. There is also evidence suggesting homozygous deletion of *CDKN2A/B* identifies poor prognosis *IDH*mt astrocytoma [2]. This finding and the 3rd cIMPACT-NOW report underscore the diagnostic importance of the routine use of a panel that simultaneously assesses glioma relevant mutations and more copy number alterations than only 1p and 19q.

The routine use of an NGS panel still requires a critical evaluation of the clinical, radiological features and histopathological findings in the case under consideration. In our series, a mistake almost made was the diagnosis of a molecular glioblastoma in a fossa posterior tumor in a young patient with histological characteristics of a medulloblastoma (7+/10-, *TERT* +). These alterations can however also be found in medulloblastoma. Also, independent of the technique used there is always the possibility of inconclusive findings. In a few cases indeed no molecular diagnosis was obtained. In most of these cases rare brain tumors without a typical molecular profile were diagnosed histopathologically. The opposite also happened: cases in which the histopathology remained inconclusive or failed to identify tumor whereas the NGS panel yielded a clear diagnosis. The most impressive experiences were of course the 6 cases in which the pathologist was unable to positively identify tumor but in which a very characteristic mutation and/or CNA pattern associated with glioma was identified, even from biopsy samples. This has of course a major clinical impact for patients.

Early 2017 the platform has been revised and expanded, to allow detection of mutations in de *TERT* promoter, in genes important for pediatric brain tumors and other adult non-glioma brain tumors and more CNA’s (including 9p, 17) that are relevant for pediatric, adolescents and young adults. Clearly, platforms like this are a moving target, and require the reconsideration of their design with new information being reported. The diagnostic specificity also depends on the specificity of the mutations and CNA’s identified within the histological context (eg. *H3F3A* K27 M mutations have now also been identified in cases of less aggressive circumscript fossa posterior lesions [5], *BRAF* mutations are not specific for a diagnostic category).

NGS is primarily aiming at mutations and allows simultaneous assessment of copy number alterations or of fusion genes, depending on the used technology. There is an increasing interest in the use of DNA genome wide methylation based classification of central nervous system tumors, which diagnostic sensitivity and clinical usefulness has been demonstrated in a recent series [3]. Each of those more broad molecular diagnostic panels have the major advantage of assessing more than one molecular feature, resulting in more in depth diagnostics. Obviously, any new version of these diagnostic panels needs to be well validated before it is introduced into routine clinical diagnostics. Ideally, this requires the close collaboration of pathologists, molecular biologists and clinicians at all stages of that process.

Limitations of the study are the testing of selected patients, in part of tertiary referrals and on clinical indications (eg, screening for trials targeted trails with targeted agents, long term glioblastoma survivors). Also, germline DNA was not investigated, which is less of an issue in case of targeted NGS but still requires the distinction between DNA variants without clinical significance and tumorigenic mutations. Next, the criteria for molecular glioblastoma are not required by the WHO 2016 classification schema to call a glioblastoma, but were used by us to have positive molecular criteria for glioblastoma, and in some cases of histological glioblastoma these were not found. Of note, the c-IMPACT-NOW 3rd update proposes the exact same criteria for ‘molecular features of glioblastoma’. Also, typical molecular abnormalities of some entities are not covered by our panel (e.g., fusion genes like *RELA* fusion genes, relevant for supratentorial ependymoma, *BRAF-KIAA* fusion gene relevant for pilocytic astrocytoma, *FGFR* fusion genes, potentially targetable). At the period studied *TERT* promoter mutations could not be assessed with our panel, but testing for this mutation has been added to the 2017 revised version of the panel. Then, of some entities characteristic mutations are not yet identified, for these methylation analysis may be better suited (e.g., posterior fossa ependymoma).

## Conclusions

Routinely using an NGS pattern allowing the simultaneous assessment of several relevant markers is a reliable and efficient way of diagnostics in brain tumors allowing a rapid diagnosis according to the WHO 2016 classification of brain tumors. It allows clinicians to evaluate the potential options for targeted therapy and provides more specific prognostic information. The flexibility of these platforms allow them to be modified once novel scientific information becomes available or changes are made in the criteria. The recent addition of gain of chromosome 7 and loss of chromosome 10 to the WHO classification emphasize this further. Anticipating an increasing role for molecular diagnostics in brain tumors and costs for NGS to decrease, NGS and other molecular broad panel diagnostics will become part of standard diagnostics of glioma in the near future.

## Additional files

Additional file 1: **Table S1.** Histological diagnosis and the NGS diagnoses subsequently made. (DOCX 21 kb)

Additional file 2: **Figure S1a, b.** A 69 year old female developed right sided weakness, T1 weighted MR images after intravenous contrast administration (a) showed an enhacing lesion in the left frontal region . A first biopsy showed brain tissue only. A second biopsy revealed some increasse in cell density with pleiomorphic cells and reactvie astrocytes, considered atypical glial cells, possibly indicative of a glioma (b, H & E stain, 100 x magnification). Next generation sequencing of this sample showed EGFR amplification, loss of chromsome 10 and a mutation in the PTEN gene (c.464A > G; p.Y155CF). (ZIP 771 kb)

Additional file 3: **Figure S2a-c.** A 38 year old female presented with burn-out complaints and several episodes suggestive of partial seizures. MR (Fluid Attenuated Inverse Recovery) images showed a small area of increased signal intensity on T2 weighted MR images with unclear boundaries and without contrast uptake (a). The lesion was resected, histology showed some cell increase without clear evidence of tumor (b, H & E stain, 100 x magnification). IDH immunohistochemistry for the R132H mutation did not show positivity in the examined region (c). On next generation sequencing, an IDH mutation (c.395 > A;p.132H) was found and a pattern suggestive of 1p/19q codeletion. The interpretation of the copy number alterations was hampered by by the low tumor cell percentage. (ZIP 1435 kb)

## Abbreviations

CNA: copy number alterations
NGS: Next Genome Sequencing
WHO: Worlth health organisation
