# Supplementary material for: Clinical evaluation of a dedicated next generation sequencing panel for routine glioma diagnostics
Source: Acta Neuropathol Commun. 2018 Nov 23;6:126. doi: 10.1186/s40478-018-0633-y (PMC6251173; doi:10.1186/s40478-018-0633-y)
Supplement: Supplementary file 1 — Table S1. Histological diagnosis and the NGS diagnoses subsequently made. (DOCX 21 kb) [file 40478_2018_633_MOESM1_ESM.docx]

**SUPPLEMENTAL FILES**

1. **SUPPLEMENTAL TABLE 1**
2. **SUPPLEMENTAL FIGURES**
   1. **Figure 1 a-b**
   2. **Figure 2 a - c**

**Supplemental table 1**

| **Histological diagnosis** |  | **Molecular diagnosis** | **Number of cases (%)** |
| --- | --- | --- | --- |
| Inconclusive |  | None | 4 (18.2) |
|  |  | Unclassifying mutations | 1 (4.5) |
|  |  | Astrocytoma | 2 (9.1) |
|  |  | Oligodendroglioma | 3 (13.6) |
|  |  | Glioblastoma | 11 (50.0) |
|  |  | *BRAF-*mutated tumor | 1 (4.5) |
|  |  | **Total** | **22 (100)** |
| Astrocytoma |  | None | 3 (3.6) |
|  |  | Unclassifying mutations | 5 (6.0) |
|  |  | Astrocytoma | 47 (56.6) |
|  |  | Oligodendroglioma | 10 (12.0) |
|  |  | Glioblastoma | 14 (16.9) |
|  |  | *H3F3A* K27M-mutated tumor | 1 (1.2) |
|  |  | *BRAF*-mutated tumor | 3 (3.6) |
|  |  | **Total** | **83 (100)** |
| Anaplastic astrocytoma |  | Unclassifying mutations | 4 (10.5) |
|  |  | Astrocytoma | 20 (52.6) |
|  |  | Glioblastoma | 9 (23.7) |
|  |  | *H3F3A* K27M-mutated tumor | 2 (5.3) |
|  |  | *H3F3A* G34M-mutated tumors | 1 (2.6) |
|  |  | *BRAF*-mutated tumor | 2 (5.3) |
|  |  | **Total** | **38 (100)** |
| Oligoastrocytoma |  | Astrocytoma | 3 (60.0) |
|  |  | Oligodendroglioma | 2 (40.0) |
|  |  | **Total** | **5 (100)** |
| Anaplastic oligoastrocytoma |  | Astrocytoma | 4 (66.7) |
|  |  | Oligodendroglioma | 1 (16.7) |
|  |  | Glioblastoma | 1 (16.7) |
|  |  | **Total** | **6 (100)** |
| Oligodendroglioma |  | None | 1 (3.3) |
|  |  | Unclassifying mutations | 2 (6.7) |
|  |  | Astrocytoma | 4 (13.3) |
|  |  | Oligodendroglioma | 22 (73.3) |
|  |  | Glioblastoma | 1 (3.3) |
|  |  | **Total** | **30 (100)** |
| Anaplastic oligodendroglioma |  | Unclassifying mutations | 1 (7.1) |
|  |  | Astrocytoma | 2 (14.3) |
|  |  | Oligodendroglioma | 11 (78.6) |
|  |  | **Total** | **14 (100)** |
| Glioblastoma |  | Unclassifying mutations | 14 (7.0) |
|  |  | Astrocytoma | 34 (16.9) |
|  |  | Oligodendroglioma | 3 (1.5) |
|  |  | Glioblastoma | 138 (68.7) |
|  |  | *H3F3A* K27M-mutated tumor | 5 (2.5) |
|  |  | *H3F3A* G34M-mutated tumors | 2 (1.0) |
|  |  | BRAF-mutated tumor | 5 (2.5) |
|  |  | **Total** | **201 (100)** |
| Ganglioglioma |  | None | 1 (16.7) |
|  |  | Unclassifying mutations | 1 (16.7) |
|  |  | Glioblastoma | 2 (33.3) |
|  |  | *BRAF*-mutated tumor | 2 (33.3) |
|  |  | **Total** | **6 (100)** |
| Pilocytic astrocytoma |  | None | 3 (33.3) |
|  |  | Unclassifying mutations | 4 (44.4) |
|  |  | *BRAF*-mutated tumor | 2 (22.2) |
|  |  | **Total** | **9 (100)** |
| Other |  | None | 7 (36.8) |
|  |  | Unclassifying mutations | 5 (26.3) |
|  |  | Astrocytoma | 1 (5.3) |
|  |  | Oligodendroglioma | 2 (10.5) |
|  |  | Glioblastoma | 3 (15.8) |
|  |  | *H3F3A* K27M-mutated tumor | 1 (5.3) |
|  |  | **Total** | **19 (100)** |

Supplemental figures.

figure 1 a, b. A 69 year old female developed right sided weakness, T1 weighted MR images after intravenous contrast administration (a) showed an enhacing lesion in the left frontal region . A first biopsy showed brain tissue only. A second biopsy revealed some increasse in cell density with pleiomorphic cells and reactvie astrocytes, considered atypical glial cells, possibly indicative of a glioma (b, H & E stain, 100 x magnification). Next generation sequencing of this sample showed EGFR amplification, loss of chromsome 10 and a mutation in the PTEN gene (c.464A>G; p.Y155CF)

figure 2 a – c

A 38 year old female presented with burn-out complaints and several episodes suggestive of partial seizures. MR (Fluid Attenuated Inverse Recovery) images showed a small area of increased signal intensity on T2 weighted MR images with unclear boundaries and without contrast uptake (a). The lesion was resected, histology showed some cell increase without clear evidence of tumor (b, H & E stain, 100 x magnification). IDH immunohistochemistry for the R132H mutation did not show positivity in the examined region (c). On next generation sequencing, an IDH mutation (c.395>A;p.132H) was found and a pattern suggestive of 1p/19q codeletion. The interpretation of the copy number alterations was hampered by by the low tumor cell percentage.
