# Supplementary figures and images for: Clinical evaluation of a dedicated next generation sequencing panel for routine glioma diagnostics
Source: Acta Neuropathol Commun. 2018 Nov 23;6:126. doi: 10.1186/s40478-018-0633-y (PMC6251173; doi:10.1186/s40478-018-0633-y)

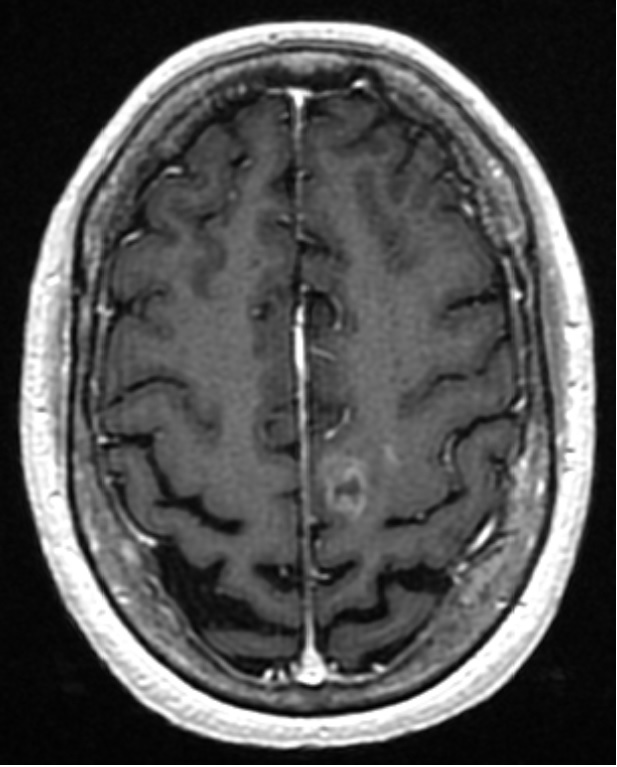

Supplement: Supplementary file 2 — Figure S1a, b. A 69 year old female developed right sided weakness, T1 weighted MR images after intravenous contrast administration (a) showed an enhacing lesion in the left frontal region . A first biopsy showed brain tissue only. A second biopsy revealed some increasse in cell density with pleiomorphic cells and reactvie astrocytes, considered atypical glial cells, possibly indicative of a glioma (b, H & E stain, 100 x magnification). Next generation sequencing of this sample showed EGFR amplification, loss of chromsome 10 and a mutation in the PTEN gene (c.464A > G; p.Y155CF). (ZIP 771 kb) [file 40478_2018_633_MOESM2_ESM.zip › suppl figure 1a.jpg]

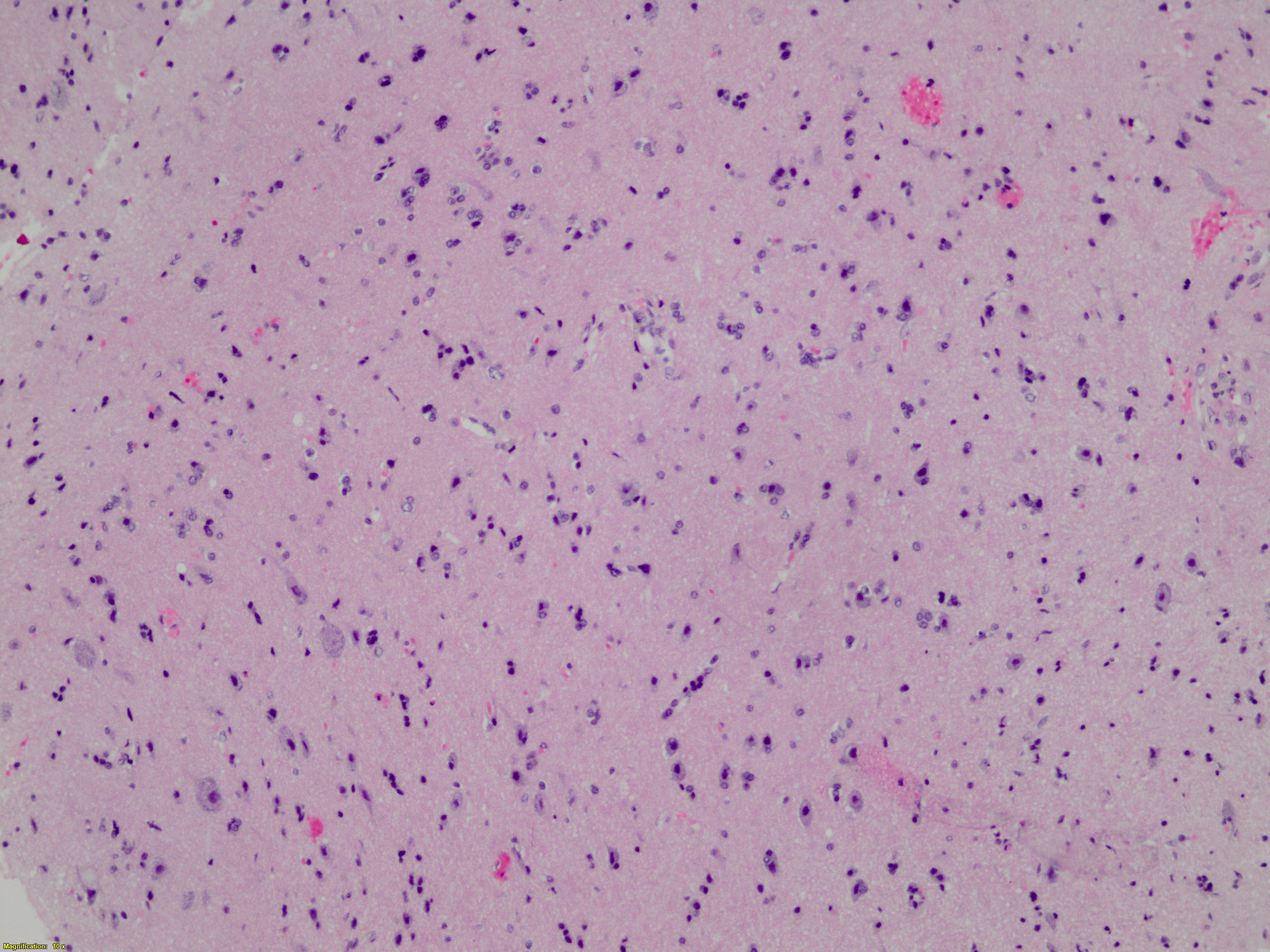

Supplement: Supplementary file 2 — Figure S1a, b. A 69 year old female developed right sided weakness, T1 weighted MR images after intravenous contrast administration (a) showed an enhacing lesion in the left frontal region . A first biopsy showed brain tissue only. A second biopsy revealed some increasse in cell density with pleiomorphic cells and reactvie astrocytes, considered atypical glial cells, possibly indicative of a glioma (b, H & E stain, 100 x magnification). Next generation sequencing of this sample showed EGFR amplification, loss of chromsome 10 and a mutation in the PTEN gene (c.464A > G; p.Y155CF). (ZIP 771 kb) [file 40478_2018_633_MOESM2_ESM.zip › suppl figure 1b.jpg]

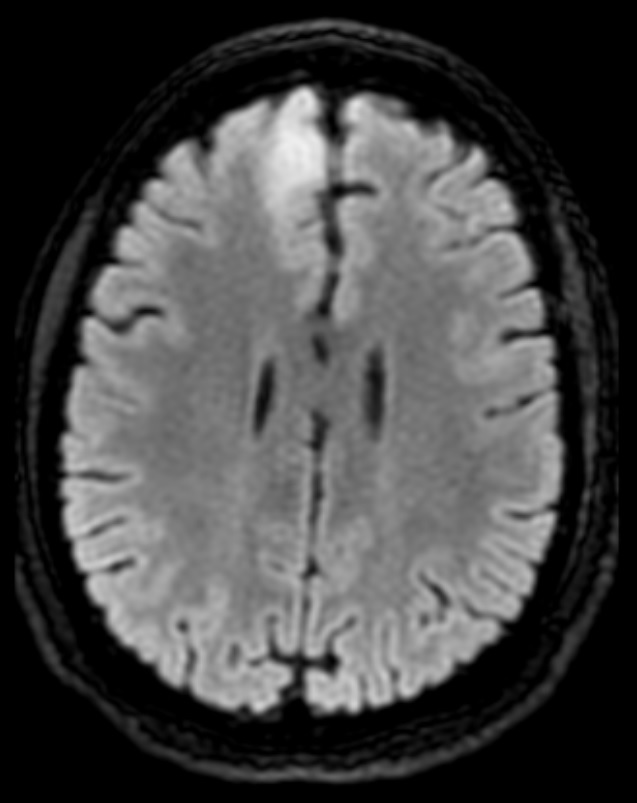

Supplement: Supplementary file 3 — Figure S2a-c. A 38 year old female presented with burn-out complaints and several episodes suggestive of partial seizures. MR (Fluid Attenuated Inverse Recovery) images showed a small area of increased signal intensity on T2 weighted MR images with unclear boundaries and without contrast uptake (a). The lesion was resected, histology showed some cell increase without clear evidence of tumor (b, H & E stain, 100 x magnification). IDH immunohistochemistry for the R132H mutation did not show positivity in the examined region (c). On next generation sequencing, an IDH mutation (c.395 > A;p.132H) was found and a pattern suggestive of 1p/19q codeletion. The interpretation of the copy number alterations was hampered by by the low tumor cell percentage. (ZIP 1435 kb) [file 40478_2018_633_MOESM3_ESM.zip › suppl figure 2a.jpg]

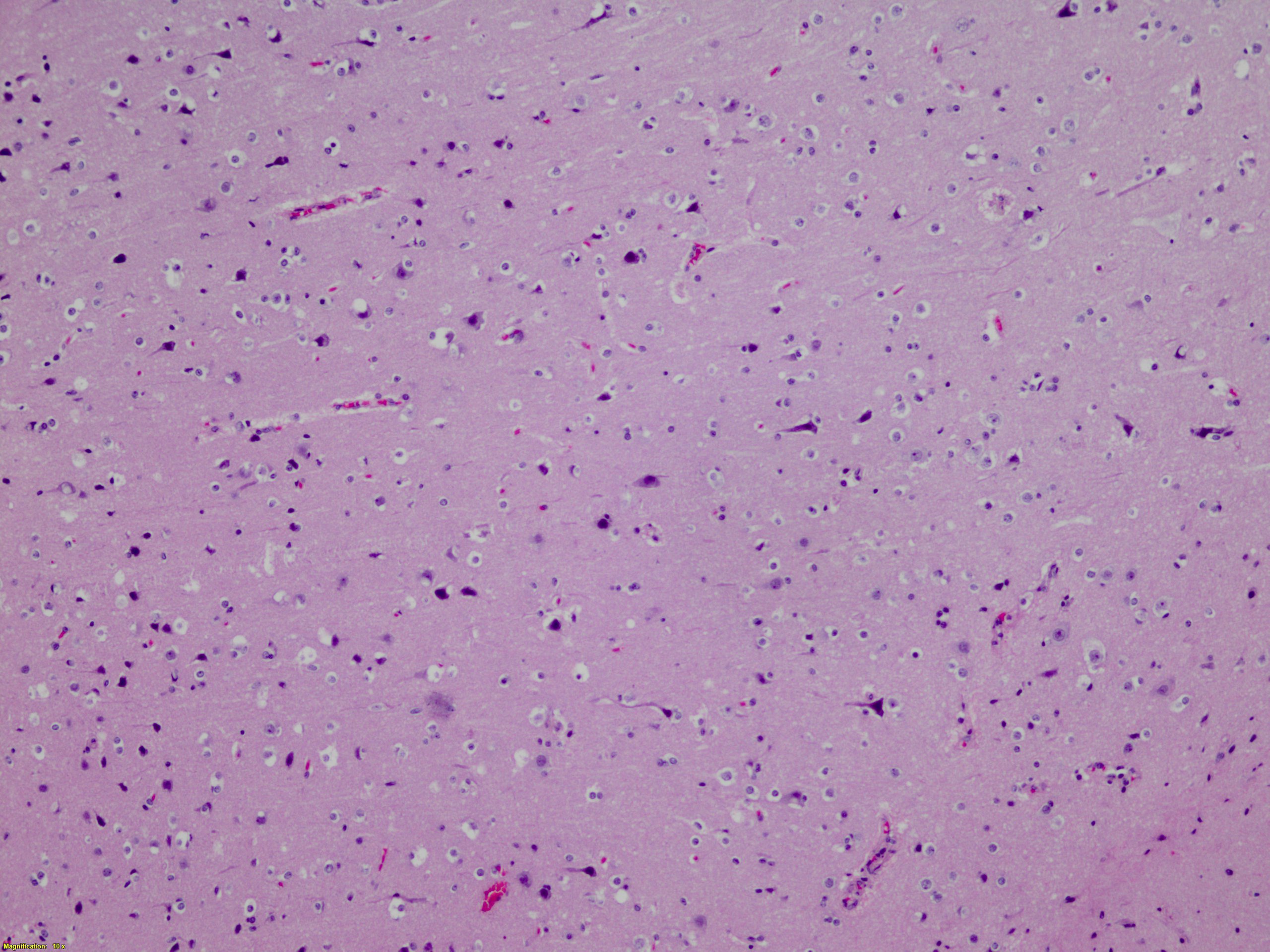

Supplement: Supplementary file 3 — Figure S2a-c. A 38 year old female presented with burn-out complaints and several episodes suggestive of partial seizures. MR (Fluid Attenuated Inverse Recovery) images showed a small area of increased signal intensity on T2 weighted MR images with unclear boundaries and without contrast uptake (a). The lesion was resected, histology showed some cell increase without clear evidence of tumor (b, H & E stain, 100 x magnification). IDH immunohistochemistry for the R132H mutation did not show positivity in the examined region (c). On next generation sequencing, an IDH mutation (c.395 > A;p.132H) was found and a pattern suggestive of 1p/19q codeletion. The interpretation of the copy number alterations was hampered by by the low tumor cell percentage. (ZIP 1435 kb) [file 40478_2018_633_MOESM3_ESM.zip › suppl figure 2b.jpg]

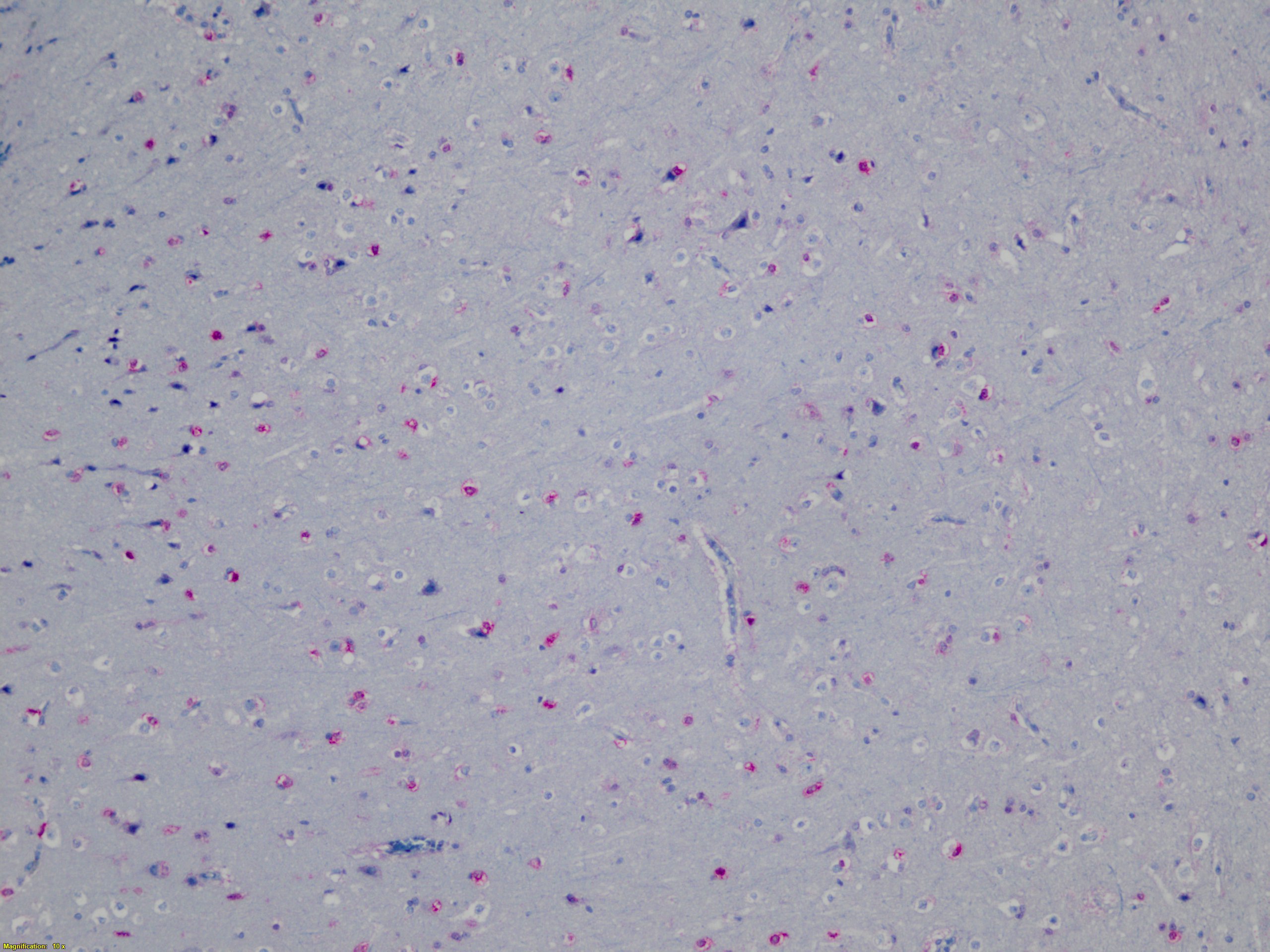

Supplement: Supplementary file 3 — Figure S2a-c. A 38 year old female presented with burn-out complaints and several episodes suggestive of partial seizures. MR (Fluid Attenuated Inverse Recovery) images showed a small area of increased signal intensity on T2 weighted MR images with unclear boundaries and without contrast uptake (a). The lesion was resected, histology showed some cell increase without clear evidence of tumor (b, H & E stain, 100 x magnification). IDH immunohistochemistry for the R132H mutation did not show positivity in the examined region (c). On next generation sequencing, an IDH mutation (c.395 > A;p.132H) was found and a pattern suggestive of 1p/19q codeletion. The interpretation of the copy number alterations was hampered by by the low tumor cell percentage. (ZIP 1435 kb) [file 40478_2018_633_MOESM3_ESM.zip › suppl figure 2c.jpg]
